# Antecedent and persistent symptoms in COVID-19 and other respiratory illnesses: Insights from prospectively collected data in the BRACE trial

**DOI:** 10.1016/j.jinf.2024.106267

**Published:** 2024-11

**Authors:** Ellie McDonald, Laure F. Pittet, Simone E. Barry, Marc Bonten, John Campbell, Julio Croda, Mariana G. Croda, Margareth Pretti Dalcolmo, Andrew Davidson, Fernando F. de Almeida e Val, Glauce dos Santos, Kaya Gardiner, Grace Gell, Amanda Gwee, Ann Krastev, Marcus Vinícius Guimaraes Lacerda, Michaela Lucas, David J. Lynn, Laurens Manning, Nick McPhate, Kirsten P. Perrett, Jeffrey J. Post, Cristina Prat-Aymerich, Lynne E. Quinn, Peter C. Richmond, Nicholas J. Wood, Nicole L. Messina, Nigel Curtis, the BRACE Trial Consortium Group

**Affiliations:** a Infectious Diseases Group, Murdoch Children's Research Institute, Parkville, Victoria, Australia; b Department of Paediatrics, The University of Melbourne, Parkville, Victoria, Australia; c Immunology, Vaccinology, Rheumatology and Infectious Diseases Unit, Geneva University Hospitals and Faculty of Medicine, Geneva, Switzerland; d Department of Thoracic Medicine, Royal Adelaide Hospital, Adelaide, South Australia, Australia; e Precision Medicine Theme, South Australian Health and Medical Research Institute, Adelaide, South Australia, Australia; f ECRAID, European Clinical Research Alliance on Infectious Diseases, Utrecht, the Netherlands; g Julius Center for Health Sciences and Primary Care, University Medical Centre Utrecht, Utrecht University, the Netherlands; h Exeter Collaboration for Academic Primary Care, University of Exeter Medical School, Exeter, United Kingdom; i Department of Epidemiology of Microbial Diseases, Yale School of Public Health, New Haven, CT, USA; j Fiocruz Mato Grosso do Sul, Fundação Oswaldo Cruz, Campo Grande, Brazil; k Universidade Federal de Mato Grosso do Sul, Campo Grande, Brazil; l Infectious Diseases, Royal Children's Hospital Melbourne, Parkville, Victoria, Australia; m Centro de Referência Professor Hélio Fraga, ENSP/FIOCRUZ (Fundação Oswaldo Cruz), Rio de Janeiro, Brazil; n Melbourne Children's Trial Centre, Murdoch Children's Research Institute, Parkville, Victoria, Australia; o Tropical Medicine Foundation Dr Heitor Vieira Dourado, Brazil; p Research Operations, The Royal Children's Hospital Melbourne, Parkville, Victoria, Australia; q Antimicrobials Group, Murdoch Children's Research Institute, Parkville, Victoria, Australia; r Fundação de Medicina Tropical Dr Heitor Vieira Dourado, Manaus, Brazil; s Instituto Leônidas & Maria Deane, Oswaldo Cruz Foundation Ministry of Health, Manaus, Brazil; t University of Texas Medical Branch, Galveston, TX, USA; u Department of Immunology, Pathwest, Queen Elizabeth II Medical Centre, Nedlands, Western Australia, Australia; v Department of Immunology, Perth Children's Hospital, Nedlands, Western Australia, Australia; w Department of Immunology, Sir Charles Gairdner Hospital, Nedlands, Western Australia, Australia; x School of Medicine, University of Western Australia, Perth, Western Australia, Australia; y Flinders Health and Medical Research Institute, Flinders University, Bedford Park, South Australia, Australia; z Department of Infectious Diseases, Fiona Stanley Hospital, Murdoch, Western Australia, Australia; aa Wesfarmers Centre for Vaccines and Infectious Diseases, Telethon Kids Institute, Nedlands, Western Australia, Australia; ab Department of Allergy and Immunology, Royal Children's Hospital Melbourne, Parkville, Victoria, Australia; ac Population Allergy Group, Murdoch Children's Research Institute, Parkville, Victoria, Australia; ad Department of Infectious Diseases, Prince of Wales Hospital, Randwick, New South Wales, Australia; ae School of Clinical Medicine, University of New South Wales, Sydney, New South Wales, Australia; af Exeter Clinical Trials Unit, Faculty of Health and Life Sciences, University of Exeter, St Luke's Campus, Heavitreee Road, Exeter, UK; ag Department of Immunology and General Paediatrics, Perth Children's Hospital, Nedlands, Western Australia, Australia; ah Faculty of Medicine and Health, University of Sydney, Sydney, New South Wales, Australia; ai National Centre for Immunisation Research and Surveillance of Vaccine Preventable Disease, Westmead, New South Wales, Australia; aj Sydney Children's Hospital Network, Westmead, New South Wales, Australia

**Keywords:** Post-acute COVID-19 syndrome (PACS), Persistent symptoms, Long COVID, COVID-19, Non-COVID-19 respiratory illness, Symptom severity, Symptom duration, Age, Chronic respiratory disease, Pre-existing symptoms, Healthcare workers, Symptom patterns, Prospective data, Multicentre study

## Abstract

**Background:**

Some individuals have a persistence of symptoms following both COVID-19 (post-acute COVID-19 syndrome; PACS) and other viral infections. This study used prospectively collected data from an international trial to compare symptoms following COVID-19 and non-COVID-19 respiratory illness, to identify factors associated with the risk of PACS, and to explore symptom patterns before and after COVID-19 and non-COVID-19 respiratory illnesses.

**Methods:**

Data from a multicentre randomised controlled trial (BRACE trial) involving healthcare workers across four countries were analysed. Symptom data were prospectively collected over 12 months, allowing detailed characterisation of symptom patterns. Participants with COVID-19 and non-COVID-19 respiratory illness episodes were compared, focussing on symptom severity, duration (including PACS using NICE and WHO definitions), and pre-existing symptoms.

**Findings:**

Compared to those with a non-COVID-19 illness, participants with COVID-19 had significantly more severe illness (OR 7·4, 95%CI 5·6–9·7). Symptom duration meeting PACS definitions occurred in a higher proportion of COVID-19 cases than non-COVID-19 respiratory controls using both the NICE definition (2·5% vs 0·5%, OR 6·6, 95%CI 2·4–18·3) and the WHO definition (8·8% vs 3·7%, OR 2·5, 95%CI 1·4–4·3). When considering only participants with COVID-19, age 40-59 years (aOR 2·8, 95%CI 1·3–6·2), chronic respiratory disease (aOR 5·5, 95%CI 1·3–23·1), and pre-existing symptoms (aOR 3·0, 95%CI 1·4–6·3) were associated with an increased risk of developing PACS. Symptoms associated with PACS were also reported by participants in the months preceding their COVID-19 or non-COVID-19 respiratory illnesses (32% fatigue and muscle ache, 11% intermittent cough and shortness of breath).

**Interpretation:**

Healthcare workers with COVID-19 were more likely to have severe and longer-lasting symptoms than those with a non-COVID-19 respiratory illness, with a higher proportion meeting the WHO or NICE definitions of PACS. Age, chronic respiratory disease, and pre-existing symptoms increased the risk of developing PACS following COVID-19.

**Research in Context**

**Evidence before this study**

Many studies of long COVID-19 have been limited by methodological issues. Ascertainment of cases, and therefore symptom data collection, has often started only after the emergence of persistent symptoms or the SARS-CoV-2 infection itself, and therefore vulnerable to selection bias and potential issues with retrospective recall. In prospective studies, methodological issues have included the accuracy of SARS-CoV-2 testing data or COVID-19 illness onset timing. Reliance on a single report of SARS-CoV-2 infection for allocation to case versus control groupings is similarly problematic, with the potential complication of subsequent infection.

**Added value of this study**

Using robust, prospectively collected data and rigorous testing for SARS-CoV-2 at multiple time points, participants with COVID-19 were found to have an increased risk of more severe and longer duration illness compared with participants who reported a non-COVID-19 respiratory illness. In participants with a COVID-19 episode, there was an increased risk of developing post-acute COVID-19 syndrome (PACS) in those aged 40–59 years, those with chronic respiratory disease and/or those with pre-existing symptoms.

**Implications of all the available evidence**

This study confirms the severity and increased duration of COVID-19 illness compared to non-COVID-19 respiratory illness. Consideration of the association between pre-existing symptoms and PACS is important for understanding the aetiology and pathophysiology of PACS, and managing patients with ongoing symptoms.

## Background

The persistence of symptoms following recovery from an acute infection is a well-established phenomenon observed in various infectious diseases.^1^, ^2^ By early July 2020, emerging evidence indicated the persistence of COVID-19 symptoms in some individuals after the acute phase of the infection (‘long COVID’).^3^ There is no universally accepted name or definition for the cluster of symptoms reported post-acute COVID-19, despite efforts made by the World Health Organization (WHO) and UK National Institute for Health and Care Excellence (NICE).^4^, ^5^, ^6^

In 2021, the WHO introduced a definition for post-acute COVID-19 syndrome (PACS) through Delphia consensus, identifying it as symptoms typically emerging three months post-initial COVID-19 onset and lasting at least two months without an alternative diagnosis. Symptoms of PACS include fatigue, shortness of breath, cough, changes in taste and smell and cognitive dysfunction, varying in intensity and potentially fluctuating or recurring over time.^6^ However, operationalising this definition in practice is challenging.^2^, ^7^ Conversely, the UK NICE framework categorises COVID-19-related symptoms into three phases, with post-COVID-19 syndrome (similar to PACS) identified by symptoms persisting beyond 12 weeks, featuring fluctuating symptom clusters affecting any body system, without a fixed symptom length or pattern.^5^ The WHO and NICE definitions differ, particularly in how they quantify persistent symptoms' duration and behaviour.

Many studies of PACS have relied on the ascertainment of cases through retrospective self-reporting of symptoms,^8^ often self-selected through support groups and social media.^9^ Other studies have enrolled participants following COVID-19 diagnosis, therefore prospective for PACS, but acknowledge the possibility of differential recruitment given a long enrolment window and potential problems with retrospective recall.^10^, ^11^, ^12^ Notable studies collected data prior to COVID-19 illness,^13^ and/or provided case-control matching with COVID-19 negative^13^ or influenza-positive controls.^14^

Using robust prospectively collected data from participants in four countries in an international trial, this study aimed 1) to compare the severity and duration of symptoms between participants with COVID-19 and those with a non-COVID-19 respiratory illness; 2) to describe the factors associated with the development of PACS following COVID-19, and 3) using COVID-19 cases and matched non-COVID-19 respiratory controls, to investigate the relationship between symptoms prior to and during the index illness with those after the illness episode.

## Methods

### Design and setting

The BRACE trial (NCT04327206) is a phase 3 multicentre randomised controlled trial (RCT) evaluating the impact of BCG vaccination on healthcare workers on COVID-19. Healthcare workers were recruited from Australia, Brazil, the Netherlands, Spain, and the United Kingdom between March 2020 and April 2021, and followed up intensively for one year. The trial protocol, including its inclusion criteria have been described previously.^15^, ^16^

### Data collection

Symptom data were collected weekly for 12 months, either through a smartphone application or by phone contact. Data were collected and managed using REDCap electronic data capture tools hosted at Murdoch Children’s Research Institute.^17^, ^18^ Prospectively collected symptoms included fever, intermittent cough, persistent cough, shortness of breath, sore throat, runny nose, headache, fatigue, loss of taste and/or smell, muscle ache, vomiting, and/or diarrhoea.^19^ When a participant reported symptoms, they were followed up daily (by smartphone reminder or phone call) until they reported resolution of the illness. An episode of illness was defined as starting on the day of the first report of symptoms and ending on the day the participant reported no longer experiencing any symptoms.

On reporting any febrile/respiratory symptom (fever, cough, shortness of breath, or sore throat), participants were requested to have a SARS-CoV-2 polymerase chain reaction (PCR) test or rapid antigen test (RAT). In addition, venous blood was collected every three months and tested for SARS-CoV-2 nucleocapsid antibodies. Quarterly surveys were used to ascertain completeness and accuracy of daily data collection (after 3, 6, 9, and 12 months of follow-up). Procedures relating to the follow-up of daily symptoms were consistent between SARS-CoV-2 PCR/RAT positive episodes and PCR-negative or untested episodes of illness.

### Study participants

**Fig. 1:**
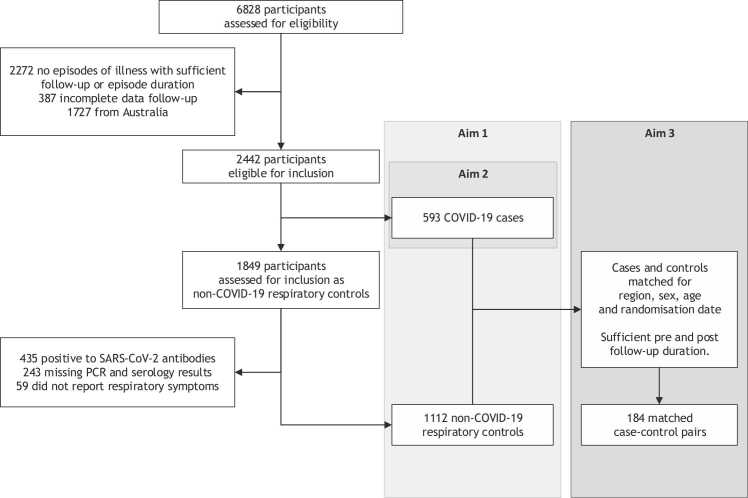
Participant inclusion flowchart.

Participants who did not have full quarterly survey completion or who did not report episodes of illness with three or more days duration over a sufficient follow-up period were excluded *a priori* from analyses. Different follow-up periods were required for the different definitions of PACS (Supplementary Table 1). Australian participants were also excluded as this country had negligible SARS-CoV-2 exposure during the trial period (Fig. 1).

A COVID-19 case was defined as a participant who reported an episode of illness with at least three days of any of the 11 symptoms, during which they tested positive for SARS-CoV-2 using PCR or RAT, and reported no subsequent SARS-CoV-2 infections during our post-illness episode observation period. Non-COVID-19 respiratory controls included all participants who had at least one episode of illness reporting respiratory symptoms, during which they reported a PCR-negative test, as well as negative SARS-CoV-2 serology and no positive PCR or RAT tests throughout the study period. Where non-COVID-19 respiratory controls had multiple episodes, the longest episode was selected as the index case. Both COVID-19 cases and non-COVID-19 respiratory controls were included only if there was a sufficient follow-up period (defined as at least 12 weeks) after the index illness. A severe episode was defined as one with three or more consecutive days where the participant reported being too sick to work (mild severity), three or more consecutive days confined to bed (moderate severity) or any hospitalisation (high severity).

A subsample of participants was selected for case-control analyses of daily symptom data, with extended pre- and post-illness episode observation periods. These comprised 184 COVID-19 cases matched on region, sex, age category and randomisation date with 184 non-COVID-19 respiratory controls. The extended observation periods were defined as: Period 1 (pre): day −84 to −1 (i.e., the 84 days (12 weeks) prior to the onset of the index illness); Period 2 (during): day 0 to 84 (weeks 0 to 12 inclusive); and Period 3 (post): day 85 to 168 (weeks 13 to 24 inclusive).

### Statistical analysis

Data were analysed using Stata version 18.^20^ Statistical methodologies are described by aims.

#### Aim 1 Comparison of severity and duration of symptoms between participants with COVID-19 and those with a non-COVID-19 respiratory illness

Univariable logistic regression was used to investigate the association between the type of illness (COVID-19 cases versus non-COVID-19 respiratory controls) and episode duration, severity, and pre-existing symptoms. Episode duration is characterised by both the NICE^5^ and WHO^6^ definitions of PACS, with application to both COVID-19 cases and non-COVID-19 respiratory controls to enable comparison.

#### Aim 2 Factors associated with the development of PACS following COVID-19

Within the COVID-19 cases, univariable and multivariable analyses were used to investigate the association between demographic and co-morbidity factors and PACS, as defined by NICE and/or WHO. Region, age, and sex were included *a priori* in the multivariable model, with any additional factors (baseline comorbidities, COVID-19 vaccination, symptoms prior to and severity of the COVID-19 episode) included if significantly associated at the univariable level.

#### Aim 3 Matched case-control analyses comparing COVID-19 and non-COVID-19 respiratory illness

To investigate the relationship between symptoms prior to and during the index illness with those after the illness episode in COVID-19 cases and matched non-COVID-19 respiratory controls, daily symptom data were categorised into Period 1 (pre), Period 2 (during) and Period 3 (post) with respect to the first day (day 0) of the COVID-19 (case) or non-COVID-19 respiratory (control) episode of illness and comprised three sub-analyses.

In the first descriptive sub-analysis (*3a*), median days and interquartile ranges of each symptom were calculated for each Period by cases and controls split into those who met either the WHO or NICE definition of PACS and those who did not. The median count of different symptoms plus the interquartile range were calculated across each Period. In addition, the count of individual symptom days across each Period for each participant who met the definition for PACS was presented as a heatmap.

In the second descriptive sub-analysis (*3b*), the total number of symptoms reported by each participant were summed across each Period and reported graphically for respiratory (intermittent cough, persistent cough, shortness of breath, sore throat, runny nose) and other symptoms (fever, headache, muscle ache, loss of taste and/or smell, vomiting and/or diarrhoea) groups.

**Fig. 2:**
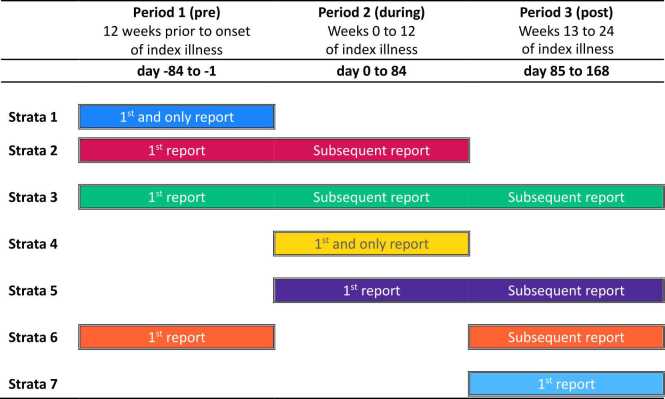
Stratification of the first and subsequent report of daily symptom data.

In the third descriptive sub-analysis (*3c*), reports of each symptom were summed by day and stratified by Periods in which the first and subsequent reports occurred. ‘Yes’ responses to any symptom on any given day during Periods 1, 2 and 3 were collapsed into seven different strata. A detailed description of the Periods each stratum encompasses is provided in Fig. 2. All descriptive sub-analysis data for respiratory and other symptom groups were presented graphically.

## Results

Of the 6828 participants in the BRACE trial, 2442 were eligible for inclusion in this study. After the exclusion of 737 participants in the non-COVID-19 group due to positive SARS-CoV-2 antibodies, lack of data on PCR and serology tests, and loss of follow-up, a total of 593 eligible participants had a COVID-19 episode and 1112 had a non-COVID-19 respiratory episode of illness that were included for analysis (Fig. 1). Baseline characteristics, including age, sex, and comorbidities, were similar between participants who had COVID-19 and those who had non-COVID-19 respiratory illness (Table 1). Participants who had COVID-19 were more likely to have been recruited in South America than those who had non-COVID-19 respiratory illness (Table 1).

**Table 1:** Baseline characteristics in COVID-19 cases and non-COVID-19 respiratory controls.

	Non-COVID-19 respiratory controls (n = 1112) n (%)	COVID-19 cases (n = 593) n (%)
Region		
Europe	387 (34·8)	104 (17·5)
South America	725 (65·2)	489 (82·5)
Age		
<40 years	512 (46·0)	279 (47·0)
40 to 59 years	517 (46·5)	279 (47·0)
≥60 years	83 (7·5)	35 (6·0)
Sex		
Male	270 (24·3)	140 (23·6)
Female	842 (75·7)	452 (76·2)
Unspecified	0 (0·0)	1 (0·2)
Diabetes		
No	1077 (96·9)	570 (96·1)
Yes	35 (3·1)	23 (3·9)
Cardiovascular disease		
No	966 (86·9)	515 (86·8)
Yes	146 (13·1)	78 (13·2)
Chronic respiratory disease		
No	1040 (93·5)	564 (95·1)
Yes	72 (6·5)	29 (4·9)
Obesity		
BMI <30 kg/m^2^	845 (76·0)	435 (74·3)
BMI ≥30 kg/m^2^	243 (22·0)	150 (25·3)
Missing	24 (2·0)	8 (1·4)
COVID-19 vaccination >14d prior to illness		
No dose prior to illness	435 (39·1)	279 (47·1)
ChAdOx1-S (*Oxford-AstraZeneca*)	262 (23·6)	115 (19·4)
BNT162b2 (*Pfizer-BioNTech*)	104 (9·4)	28 (4·7)
mRNA-1273 (*Moderna*)	16 (1·4)	3 (0·5)
CoronaVac (*Sinovac*)	287 (25·8)	166 (28·0)
Ad26. COV2. S (*Johnson & Johnson*)	8 (0·7)	2 (0·3)

### Comparison of severity and duration of symptoms between participants with COVID-19 and those with a non-COVID-19 respiratory illness (Aim 1)

Severe illness occurred in a higher proportion of COVID-19 cases than non-COVID-19 respiratory controls (OR 7·4, 95%CI 5·6–9·7).

Symptom duration meeting PACS definitions occurred in a higher proportion of COVID-19 cases than non-COVID-19 respiratory controls using both the NICE definition (2·5% vs 0·5%, OR 6·6, 95%CI 2·4–18·3) or the WHO definition (8·8% vs 3·7%, OR 2·5, 95%CI 1·4–4·3) (Table 2). COVID-19 cases did not differ significantly from non-COVID-19 respiratory controls in the report of symptoms prior to the index episode of illness (Table 2). The mean duration of symptoms by demographics and comorbidities for cases and controls are shown in Supplementary Figures 1 to 7.

**Table 2:** Comparison of episode duration, pre-existing symptoms, and severity of index episode between COVID-19 cases and non-COVID-19 respiratory controls.

	Non-COVID-19 respiratory controls	COVID-19 cases	Unadjusted
	n/N (%)	n/N (%)	OR (95% CI)
Severe index episode^a^			
No	995/1112 (89·5)	317/593 (53·5)	1·0 ref
Yes	117/1112 (10·5)	276/593 (46·5)	7·4 (5·6−9·7)*
≥3 days of any symptoms in Period 1^b^			
No	450/670 (67·2)	235/364 (64·6)	1·0 ref
Yes	220/670 (32·8)	129/364 (35·4)	1·1 (0·9−1·5)
Episode duration (NICE definition)			
<4 weeks (Acute)	1078/1112 (96·9)	493/593 (83·1)	1·0 ref
4 to 12 weeks (Ongoing symptomatic)	29/1112 (2·6)	85/593 (14·3)	6·4 (4·1−10·0)*
>12 weeks (Persistent)	5/1112 (0·5)	15/593 (2·5)	6·6 (2·4−18·3)*
Meets WHO definition^c^			
No	645/670 (96·3)	332/364 (91·2)	1·0 ref
Yes	25/670 (3·7)	32/364 (8·8)	2·5 (1·4−4·3)*
Meets either NICE or WHO criteria			
No	1084/1112 (97·2)	555/593 (93·3)	1·0 ref
Yes	28/1112 (2·8)	38/593 (6·8)	2·7 (1·6−4·4)*

* p < 0·001.

a Severe: ≥3 days too sick to work or ≥3 days confined to bed or hospitalised.

b Sample limited by pre-index follow-up availability of at least 7 days.

c Symptoms begin/recur 3 months post episode start and span 2 months.

### Factors associated with PACS following COVID-19 (Aim 2)

Factors associated with PACS following COVID-19 are shown in Table 3. In the adjusted models, chronic respiratory disease was associated with PACS using the NICE definition, report of symptoms prior to the COVID-19 episode was associated with PACS using the WHO definition, and age (40–59 years) was associated with PACS using both definitions. When including participants who met either definition, age and report of symptoms prior to the index episode of COVID-19 were associated with PACS in the adjusted model. Sex, diabetes, cardiovascular disease, and obesity showed no association with either definition of PACS.

**Table 3:** Demographic factors and co-morbidities associated with COVID-19 PACS (as defined by NICE, WHO or either).

	1. Meets NICE criteria^a^			2. Meets WHO criteria^b^			3. Meets either NICE or WHO criteria		
	N (%)	UadjOR (95% CI)	aOR^c^ (95% CI)	N (%)	UadjOR (95% CI)	aOR^c^ (95% CI)	N (%)	UadjOR (95% CI)	aOR^c^ (95% CI)
**Overall**	15/593 (2·5)	n/a	n/a	32/364 (8·8)	n/a	n/a	38/593 (6·8)	n/a	n/a
**Region**									
Europe	5/104 (4·8)	1·0 ref	1·0 ref	11/74 (14·9)	1·0 ref	1·0 ref	13/104 (12·5)	1·0 ref	1·0 ref
South America	10/489 (2·0)	0·4 (0·1−1·2)	0·6 (0·2−2·0)	21/290 (7·2)	0·5 (0·2−1·0)	0·4 (0·2−1·0)	25/489 (5·1)	0·4 (0·2−0·8)**	0·5 (0·2−1·2)
**Age categories**									
<40 years	3/279 (1·1)	1·0 ref	1·0 ref	10/173 (5·8)	1·0 ref	1·0 ref	12/279 (4·3)	1·0 ref	1·0 ref
40−59 years	12/279 (4·3)	4·1 (1·2−14·9)*	4·3 (1·2−15·8)*	21/168 (12·5)	2·3 (1·1−5·1)*	2·4 (1·1−5·4)*	25/279 (9·0)	2·2 (1·1−4·5)*	2·8 (1·3−6·2)*
≥60 years	0 (0)	n/a	n/a	1/23 (4·4)	0·7 (0·1−6·1)	0·5 (0·1−4·7)	1/35 (2·9)	0·7 (0·1−5·2)	n/a
**Sex categories**									
Male	2/140 (1·4)	1·0 ref	1·0 ref	5/81 (6·2)	1·0 ref	1·0 ref	6/140 (4·3)	1·0 ref	1·0 ref
Female	13/452 (2·9)	2·0 (0·5−9·2)	2·0 (0·4−9·1)	27/283 (9·5)	1·6 (0·6−4·3)	1·4 (0·5−3·9)	32/452 (7·1)	1·7 (0·7−4·2)	1·3 (0·5−3·5)
**Diabetes**	1/23 (4·4)	1·8 (0·2−14·4)	n/a	1/15 (6·7)	0·7 (0·1−5·8)	n/a	2/23 (8·7)	1·4 (0·3−6·3)	n/a
**Cardiovascular disease**	4/78 (5·1)	2·5 (0·8−8·0)	n/a	4/41 (9·8)	1·1 (0·4−3·4)	n/a	4/78 (5·1)	0·8 (0·3−2·2)	n/a
**Respiratory disease**	3/29 (10·3)	5·3 (1·4−20·2)**	5·5 (1·3−23·1)*	4/23 (17·4)	2·4 (0·7−7·4)	n/a	5/29 (17·2)	3·4 (1·2−9·4)*	1·5 (0·4−5·4)
**Obesity** (BMI ≥30 kg/m^2^)	7/150 (4·7)	2·6 (0·9−7·4)	n/a	9/90 (10·0)	1·2 (0·5−2·7)	n/a	12/150 (8·0)	1·4 (0·7−2·9)	n/a
**COVID−19 vaccination**									
No dose prior to illness^d^	9/279 (3·2)	1·0 ref	n/a	23/224 (10·3)	1·0 ref	n/a	26/279 (9·3)	1·0 ref	1·0 ref
ChAdOx1-S (*Oxford-AstraZeneca*)	3/115 (2·6)	0·8 (0·2−3·0)		5/48 (10·4)	1·0 (0·4−2·8)		7/115 (6·1)	0·6 (0·3−1·5)	1·0 (0·3−3·1)
BNT162b2 (*Pfizer-BioNTech*)	0/28 (0)	n/a		0/5 (0)	n/a		0/28 (0)	n/a	n/a
mRNA−1273 (*Moderna*)	0/3 (0)	n/a		0/1 (0)	n/a		0/3 (0)	n/a	n/a
CoronaVac (*Sinovac*)	3/15 (20·0)	0·6 (0·2−2·1)		4/86 (4·7)	0·4 (0·1−1·3)		5/166 (3·0)	0·3 (0·1−0·8)*	0·7 (0·2−2·0)
Ad26. COV2. S (*Johnson & Johnson*)	0/2 (0)	n/a		0/0 (0)	n/a		0/0 (0)	n/a	n/a
**≥3 days of any symptoms prior to COVID−19 episode (Period 1)**^e^	7/129 (5·4)	2·6 (0·8−8·5)	n/a	19/129 (14·7)	3·0 (1·4−6·3)**	3·0 (1·4−6·3)**	19/129 (14·7)	2·4 (1·2−4·8)*	2·3 (1·1−4·9)*
**Severe COVID−19 episode**^f^	11/276 (4·0)	3·3 (1·0−10·4)*	2·7 (0·8−9·1)	14/190 (7·4)	0·7 (0·3−1·4)	n/a	20/276 (7·3)	1·3 (0·7−2·5)	n/a

* p < 0·05.

** p < 0·01.

a Consistent symptoms ≥12 weeks.

b Symptoms begin/recur 3 months post episode and span 2 months.

c Data were adjusted *a priori* for region, age and sex and variables significant at the univariable level.

d “Dose prior” refers to >14 days prior.

e Sample limited by pre-index follow-up availability.

f Severe= ≥3 consecutive days too sick to work or ≥3 consecutive days confined to bed or hospitalised.

### Matched case-control analyses comparing COVID-19 and non-COVID-19 respiratory illness (Aim 3)

#### Symptoms associated with post-acute disease syndrome (sub-analysis 3a)

Among the 15 COVID-19 cases with sufficient follow-up data who met either the NICE or WHO definitions for PACS, the most frequently reported symptoms post-acute disease (i.e., Periods 2 and 3), were headache, runny nose, and cough (Supplementary Figure 8). Median days (IQR) during Period 2 for headache was 19 days (IQR 3–40), runny nose 7 days (IQR 4–13), and cough 7 days (IQR 1–29). Median days (IQR) during Period 3 for headache was 2 days (IQR 0–12), runny nose 6 days (IQR 0–13) and cough 2 days (IQR 0–12) (Supplementary Table 2). Participants with PACS reported a median of 7 different symptoms (IQR 5–9) during Period 2, and 4 different symptoms (IQR 4–6) during Period 3 (Supplementary Table 2).

##### Individual symptom burden (sub-analysis 3b)

**Fig. 3:**
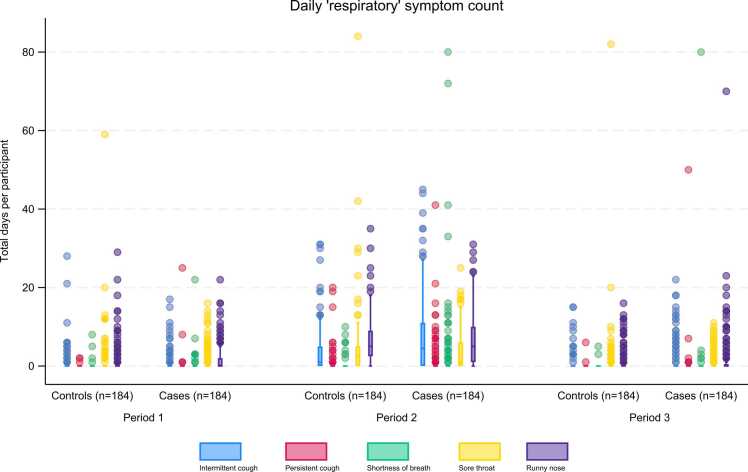
Total respiratory symptoms per participant over each period; non-COVID-19 respiratory controls compared to COVID-19 cases.

**Fig. 4:**
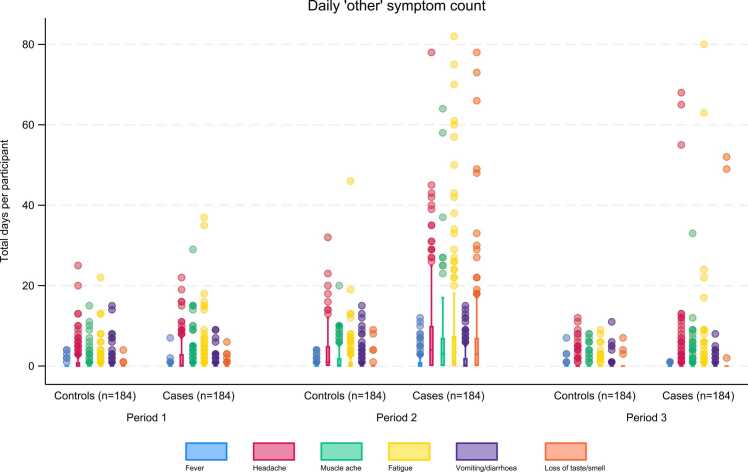
Total other symptoms per participant over each period; non-COVID-19 respiratory controls compared to COVID-19 cases.

In all three Periods, the total number of days of respiratory or other symptoms summed by participant, was greater overall for COVID-19 cases than for non-COVID-19 respiratory controls (Fig. 3, Fig. 4). This was most marked in Periods 2 and 3, particularly for fatigue and loss of taste/smell (Fig. 4).

##### Symptom prevalence across Periods (sub-analysis 3c)

**Fig. 5:**
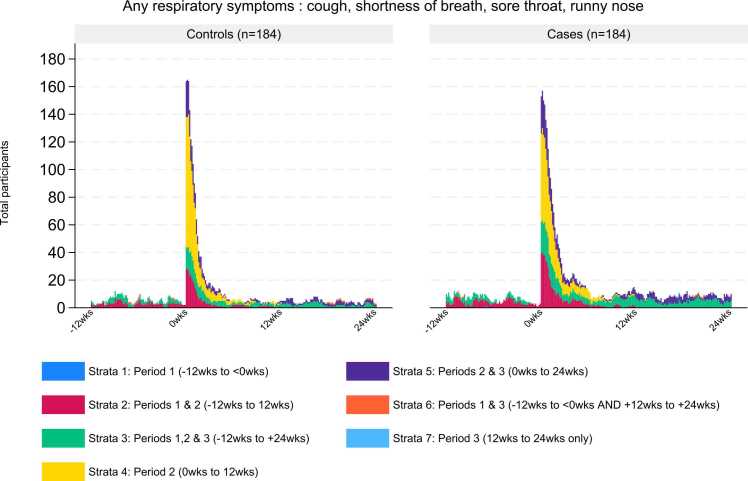
Any respiratory symptom (days) before, during and after COVID-19 and non-COVID-19 illness.

**Fig. 6:**
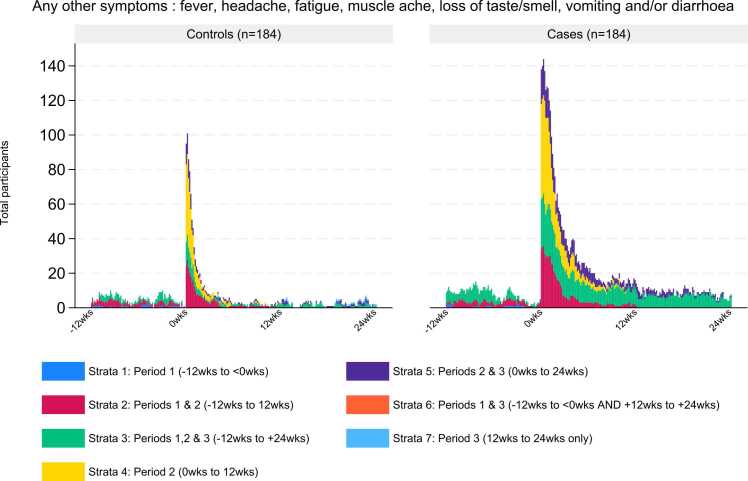
Any other symptoms (days) before during and after COVID-19 and non-COVID-19 illness.

When comparing the respiratory and other symptoms reported each day during the three Periods, the highest number of symptoms reported were in cases, during the first weeks of Period 2 (Fig. 5, Fig. 6), which correspond to the most acute phase of the illness.

The extent and duration of respiratory symptoms were similar between cases and controls, particularly in strata 4 and 5 (diagrammatic explanation of strata provided in Fig. 2), in which symptoms first occurred during Period 2 (Fig. 5). There were a higher number of cases compared to controls who had symptom onset in Period 1 (strata 2 and 3), both prior to the index illness and after. However, when tested using univariable logistic regression (for aim 1), cases did not have significantly higher odds of reporting ≥3 days of symptoms prior to the index episode (Table 2).

There was a greater range and duration of other symptoms across all strata in cases compared with controls (Fig. 6). Symptoms of fatigue, muscle ache, headache, and loss of taste and/or smell contributed most to the total symptom burden in COVID-19 cases (Supplementary Figures 9–13).

## Discussion

Our study compared the severity, pattern, and duration of symptoms in adults with COVID-19 and non-COVID-19 respiratory episodes using robust prospectively collected data. Participants with COVID-19 were seven times more likely to report a higher severity of illness than participants with non-COVID-19 respiratory episodes.^21^, ^22^ The proportion of participants reporting symptoms 12 weeks after the onset of COVID-19 was between 2·5% and 8·8% depending on the criteria used, representing a significant long-term burden of continuing symptoms.^5^, ^6^, ^23^ Notably, COVID-19 episodes were up to six times more likely to be prolonged than non-COVID-19 respiratory episodes.

Our finding is consistent with other studies that report SARS-CoV-2 is more likely than other viruses to cause long-lasting symptoms.^13^, ^22^ In contrast, a recent study reported symptoms 12 weeks after COVID-19 are no different from influenza.^24^ A possible explanation for this conflicting finding is differential inclusion in the two groups, with a bias towards severe influenza but all-severity COVID-19. Testing for influenza is targeted at those with more severe illness, whereas testing for COVID-19 has become common in the general population, regardless of health status or severity of symptoms.

Our study was unique in collecting symptom data prior to the index episodes of COVID-19 or non-COVID-19 respiratory illness. One-third of participants had symptoms in the 12 weeks prior to their index illness. In this period, those with COVID-19 reported more shortness of breath, persistent cough, muscle ache, headache, and fatigue than non-COVID-19 respiratory illness controls. Participants who reported at least three days of any symptom prior to the index episode were twice as likely to develop PACS, particularly using the WHO definition. Our study further revealed that chronic respiratory symptoms are a key factor associated with PACS as defined by NICE, consistent with previous studies.^25^ Whilst the association between pre-existing symptoms and PACS may be due to underlying co-morbidities, this finding highlights the impact of pre-existing health conditions on COVID-19 long-term outcomes.

There was a greater diversity of symptoms reported by participants with COVID-19 than by those with non-COVID-19 respiratory illness. Our finding that other symptoms (fatigue, headache, muscle ache and loss of taste/smell) were more common in COVID-19 cases than the controls is consistent with the more extensive systemic impact of COVID-19.^22^ These findings support the histopathological evidence of an exuberant immune reaction associated with SARS-CoV-2 infection.^26^ Additionally, the diversity of symptoms in the first 12 weeks of illness (Period 2) was also greater in COVID-19 cases that subsequently met any PACS definition compared to those who did not, consistent with the clinical evidence of PACS being a multi-organ disease.^9^, ^23^, ^26^, ^27^

The understanding that loss of taste and/or smell was rare for non-COVID-19 respiratory illnesses, but a common, and to some extent defining, symptom of COVID-19 is strongly supported in our study.^13^ Loss of taste and/or smell was rarely reported by participants in either group prior to the index episode of illness (Period 1). Similarly, the non-COVID-19 respiratory controls reported negligible loss of taste and/or smell during or after their episode of illness. This contrasts sharply with COVID-19 cases, in which over half reported loss of taste and/or smell during Period 2, irrespective of PACS.

The strengths of our study lie in its methodological rigour, particularly the prospective daily data collection, limiting recall and selection bias, and the meticulous ascertainment of SARS-CoV-2 infection. Including both post-episode serological testing and PCR/RAT testing during illness episodes enabled a precise distinction between cases and controls. This methodology ensured both accurate COVID-19 classification and the absence of subsequent COVID-19 illness throughout the follow-up period in the non-COVID-19 group. A further strength is the inclusion only of participants for whom data were available for the entire follow-up period. Although this reduced the number of participants included in the analysis, it ensured accurate ascertainment of cases and controls.

A limitation of this study is that the study protocol and surveys were developed when the breadth of COVID-19 symptoms was first emerging and before the existence of persistent symptoms was appreciated, including novel symptoms, such as neurocognitive impairment or psychological conditions.^11^, ^19^ Therefore, only a limited number of associated symptoms were investigated, and may not encompass all of the symptoms already described as associated with PACS, which may have reduced the number of participants included in this study and/or the number of symptoms and duration of post-acute disease.

A further limitation of our study is that we were unable to determine the type of non-COVID-19 respiratory infection in the control group, and whether this was a first exposure for each participant or a repeat exposure. Given the life-long exposure to non-COVID-19 respiratory viruses, it is likely that some of the difference between COVID-19 episodes and non-COVID-19 respiratory infections is attributable to the differential effects of first exposure compared with repeated exposure in the control group. Also, it was not possible to detect if the symptoms present in Period 3 were caused by a subsequent infection with another respiratory pathogen. However, this was likely mitigated by case-control matching by region and randomisation date, with exposure for both cases and controls likely being similar for both respiratory viruses and particular COVID-19 strains in circulation.

Our findings underline the challenge of using the WHO definition of PACS, due to the breadth of definition coupled with a proviso that the symptoms “cannot be explained by an alternative diagnosis”.^6^ This is a difficult criterion to apply in a research context without a detailed prior medical history, current medical records, and case-by-case assessment. The associations between WHO-defined PACS and the report of symptoms prior to the illness indicate the possibility of a long-term continued reporting of pre-existing baseline symptoms, unrelated to the COVID-19 episode of illness.

In conclusion, using prospectively collected data, our study provides a robust estimate of the incidence of PACS according to the current definitions. It shows that persistent symptoms are more common after COVID-19 than other non-COVID-19 respiratory illnesses, and that symptoms are common prior to the onset of an episode of COVID-19 or non-COVID-19 respiratory illness. Moreover, the association between symptoms prior to a COVID-19 episode and PACS should be considered when understanding the aetiology of long-term symptoms following COVID-19. This also highlights the importance of an improved definition of PACS for managing patients with ongoing symptoms and when investigating its pathophysiology.

## Ethics approval

The study was approved by the Royal Children’s Hospital Melbourne Human Research Ethics Committee (No.62586); the protocol was approved by the ethics committee at each site and all participants provided informed consent. The trial was overseen by a steering committee and a data safety and monitoring board.

## Funding

The trial is supported by the 10.13039/100000865Bill & Melinda Gates Foundation [INV017302], the Minderoo Foundation [COV-001], Sarah and Lachlan Murdoch, the Royal Children’s Hospital Foundation [2020–1263 BRACE Trial], Health Services Union NSW, the Peter Sowerby Foundation, SA Health, the Insurance Advisernet Foundation, the NAB Foundation, the Calvert-Jones Foundation, the Modara Pines Charitable Foundation, the UHG Foundation Pty Ltd, Epworth Healthcare and individual donors. The Murdoch Children’s Research Institute (MCRI) leads the BRACE trial across 36 sites in five countries. It is supported by the Victorian Government’s Operational Infrastructure Support Programme. NC is supported by a 10.13039/501100000925National Health and Medical Research Council (NHMRC) Investigator Grant (GNT1197117). LFP is supported by the 10.13039/501100001711Swiss National Science Foundation (Early Postdoc Mobility Grant, P2GEP3_178155).

## Role of funding source

The funders of this study had no role in study design, data collection, data analysis, data interpretation, or writing of the report.

## Authors contributions

All authors contributed substantially to the BRACE trial. NC is the chief principal investigator of the BRACE trial. EMD did all the statistical analysis with substantial input from NLM, NC, and LFP. EMD wrote the first draft of the report with input from NLM, NC, and LFP, and all authors critically reviewed it. All authors had final responsibility for the decision to submit for publication. NC attests that all listed authors meet authorship criteria and that no others meeting the criteria have been omitted.

## Declaration of Competing Interest

The authors declare that they have no known competing financial interests or personal relationships that could have appeared to influence the work reported in this paper.

## Data Availability

Deidentified participant data and data dictionary are available to others on request and on completion of a signed data access agreement. Requests can be made in writing to braceresearch@mcri.edu.au.
